# Mast Cell, the Neglected Member of the Tumor Microenvironment: Role in Breast Cancer

**DOI:** 10.1155/2018/2584243

**Published:** 2018-02-05

**Authors:** Angélica Aponte-López, Ezequiel M. Fuentes-Pananá, Daniel Cortes-Muñoz, Samira Muñoz-Cruz

**Affiliations:** ^1^Unidad de Investigación en Virología y Cáncer, Hospital Infantil de México Federico Gómez, Dr. Márquez 162, Doctores, Cuauhtémoc, 06720 Ciudad de Mexico, Mexico; ^2^Programa de Doctorado en Ciencias Biomédicas, Universidad Nacional Autónoma de México (UNAM), Ciudad de México, Mexico; ^3^Programa de Servicio Social en Investigación en Salud, Departamento de Inmunología y Reumatología, Instituto Nacional de Ciencias Médicas y Nutrición Salvador Zubirán, Vasco de Quiroga 15, Tlalpan, 14080 Ciudad de Mexico, Mexico; ^4^Unidad de Investigación Médica en Enfermedades Infecciosas y Parasitarias, Centro Médico Nacional Siglo XXI, Instituto Mexicano del Seguro Social, Avenida Cuauhtémoc 330, Doctores, Cuauhtémoc, 06720 Ciudad de Mexico, Mexico

## Abstract

Mast cells are unique tissue-resident immune cells that secrete a diverse array of biologically active compounds that can stimulate, modulate, or suppress the immune response. Although mounting evidence supports that mast cells are consistently infiltrating tumors, their role as either a driving or an opposite force for cancer progression is still controversial. Particularly, in breast cancer, their function is still under discussion. While some studies have shown a protective role, recent evidence indicates that mast cells enhance blood and lymphatic vessel formation. Interestingly, one of the most important components of the mast cell cargo, the serine protease tryptase, is a potent angiogenic factor, and elevated serum tryptase levels correlate with bad prognosis in breast cancer patients. Likewise, histamine is known to induce tumor cell proliferation and tumor growth. In agreement, mast cell depletion reduces the size of mammary tumors and metastasis in murine models that spontaneously develop breast cancer. In this review, we will discuss the evidence supporting protumoral and antitumoral roles of mast cells, emphasizing recent findings placing mast cells as important drivers of tumor progression, as well as the potential use of these cells or their mediators as therapeutic targets.

## 1. Introduction

The association between chronic inflammation and cancer has long been recognized. Inflammation evolved as part of the body's defense against internal and external stimuli that disrupt tissue homeostasis. It aims to eliminate the stimuli, repair the damaged tissue, and reestablish homeostasis. When inflammation is maintained for a short period of time, it usually comes with therapeutic consequences; however, when it is chronically sustained, it has the potential to enhance or promote the emergence of malignancies [1–3]. Virchow proposed a link between chronic inflammation and cancer as early as the 19th century, and he hypothesized that inflamed tissues were the primed sites in which cancer lesions were initiated [4]. Indeed, mounting evidence supports that chronic inflammation provides conditions that lead to malignant transformation. Immune cells persistently infiltrating tissues are actively inducing oxidative stress and releasing soluble mediators, such as cytokines, chemokines, and growth factors, which alter genes and proteins involved in cell cycle, DNA repair, and apoptosis [5, 6]. Besides initiation, chronic inflammation seems to be continually important during tumor progression, creating a favorable microenvironment that contributes to tumor cell proliferation, survival, invasion, migration, tissue remodeling, and angiogenesis, ending in cancer metastasis [7].

Epidemiological data estimate that at least one-third of all cancers are associated with chronic infections or with evident long-lasting unresolved inflammation [8, 9]. Some of the well-described infection- and inflammation-associated cancers are gastric, colorectal, cervical, and hepatocellular carcinoma [3, 10]. Breast cancer has also been associated with chronic inflammation, although the inflammatory stimulus is less clear. The stroma of breast tumors is generally enriched with a great variety of inflammatory cells, which however do not seem to be protective. Moreover, several studies indicate that tumor cells can evade the immune responses and enhance inflammation favoring cancer evolution to aggressive stages [11, 12]. Among the best characterized immune cell populations present in the stroma of breast cancers are the tumor-associated macrophages, which have been linked to cancer aggressive features, such as angiogenesis, degradation of extracellular matrix (ECM) proteins, and invasion [13]. Likewise, it has become evident that other immune cells, such as neutrophils and mast cells, are consistently found in the breast cancer stroma, most likely contributing to the inflammatory microenvironment that shapes cancer behavior [13, 14]. In this review, we will discuss the evidence supporting protumoral and antitumoral roles of mast cells in breast cancer progression.

## 2. Mast Cell Biology

Mast cells are granulated innate immune cells characterized by their cargo of inflammatory mediators, comprised of a wide array of preformed bioactive molecules stored in cytoplasmic granules, which are released upon encountering the appropriate stimuli and have beneficial roles in immunological responses against pathogens, including intestinal helminths, bacteria, and viruses. Mast cell-derived mediators also participate in tissue physiological processes, such as wound healing and tissue repair, and in some pathological conditions [15]. For instance, IgE-induced mast cell degranulation triggers the immediate hypersensitivity reactions that play a central role in the pathogenesis of allergic diseases [16].

**Figure 1:**
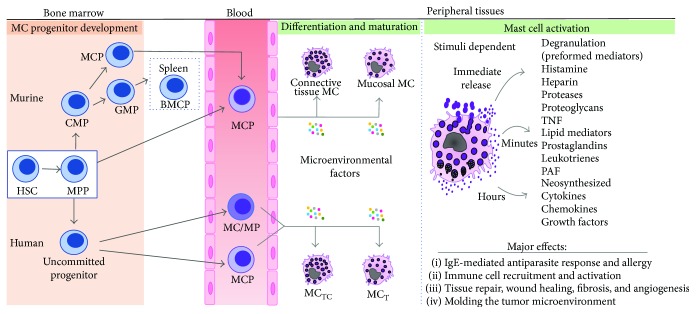
Overview of mast cell development, heterogeneity, and activation. Mast cells arise in the bone marrow from hematopoietic stem cells (HSC) via a multipotent progenitor (MPP), which can become a mast cell-committed progenitor (MCP) that exits the bone marrow and migrates to peripheral tissues to complete maturation. Several pathways have been described for murine and human mast cell origin. In mice, MCPs may be derived directly from MPPs or from common myeloid progenitors (CMP). Mast cells may also be derived from the granulocyte/monocyte progenitor (GMP) via an intermediate progenitor (BMCP), identified only in the spleen of C57BL6 mice, which gives rise to basophils and mast cells. In humans, it has been postulated that mast cells originate from a yet unidentified uncommitted progenitor that gives rise to a mast cell/monocyte-committed progenitor (MC/MP) in bone marrow. Alternatively, an MCP population that gives rise exclusively to mast cells has recently been identified in blood. Final differentiation occurs in peripheral tissues, where microenvironmental factors determine the phenotype of the mature mast cells. Mast cells exhibit marked phenotypic and functional heterogeneity. Two major subtypes have been described in both rodents and humans, in the former as mucosal and connective tissue mast cells and in the latter as tryptase- and chymase-rich mast cells (MC_TC_) and those that mainly contain tryptase (MC_T_). The right end diagram illustrates the distinct classes of bioactive molecules and their temporality of release upon mast cell activation in tissues. See text for a more detailed explanation.

Mast cells are distributed in diverse tissues throughout the body, but a considerable number of them are located close to blood vessels, nerves, and mucosal surfaces. Some of the tissues in which they are most prominent are the dermis, hypodermis, and the respiratory and gastrointestinal tract [17, 18]. Like other immune cells, mast cells originate in the bone marrow from hematopoietic stem cells via a multipotent progenitor, which can become a committed mast cell progenitor (MCP) that exits the marrow and migrates to peripheral tissues to complete maturation. Early mast cell progenitors in bone marrow do not contain cytoplasmic granules and do not express FcεRI on their surface. A slightly more differentiated MCP, identified in tissues in mice and in bone marrow in rats, contains few small cytoplasmic granules, express high levels of integrin *β*7, and can often also express the FcεRI. This seems to be the mast cell progenitor that leaves the bone marrow [19–22]. The MCPs arrive to diverse peripheral tissues by transendothelial migration in which they complete their differentiation under the control of microenvironmental cytokines and growth factors [23, 24]. Over the last years, several models for mast cell development have been proposed; however, the ontogenesis of mast cells in mice and humans is only beginning to be understood, and knowledge of the specific signals that modulate progenitor recruitment and differentiation is still limited. Mast cell development in mice and humans share some similarities but also exhibit major differences. In mice, different mast cell progenitor populations have been described depending on the particular strain [19–22]. So far, very few studies have attempted to characterize the mechanisms involved in human mast cell development [25–27]. Outstandingly, a recent study has identified a blood-derived human mast cell progenitor population that gives rise exclusively to mast cells. These cells express the FcεRI and integrin *β*7 and display a mast cell-like phenotype, although with a limited cell division capacity *in vitro* [28]. In both humans and mice, a complex network of signaling molecules and transcription factors regulates formation of MCPs in bone marrow and their migration to tissues in which they develop into fully competent mature mast cells.  Figure 1 illustrates a simplified overview of mast cell development and heterogeneity.

Mast cell differentiation, growth, and survival are strongly regulated by local tissue environmental factors. Stem cell factor (SCF), the ligand of the c-Kit receptor, and IL-3 are among the best-characterized factors. SCF is mainly secreted by fibroblasts and other mesenchymal cells and has an important role in survival, development, and expansion of mast cells [29, 30]. While, IL-3 is considered the main cytokine responsible for the T cell-induced proliferation and differentiation of mast cells, at least in rodents [31, 32]. Other endogenous factors contributing to mast cell maturation and function in rodents and humans are IL-4, IL-6, IL-9, IL-10, IL-33, nerve growth factor (NGF), and transforming growth factor-*β* (TGF-*β*) [31–34].

At least two major populations of mature mast cells have been described in humans based on their protease content. Mast cells containing only tryptase are termed MC_T_, while those containing tryptase, chymase, carboxypeptidase A, and cathepsin G are named MC_TC_. These mast cell subsets differ in their tissue localization; for instance, the MC_TC_ is the predominant type found in normal skin and small bowel submucosa, whereas the MC_T_ is almost the exclusive type found in small bowel mucosa and in bronchial/bronchiolar areas [35]. These mast cell subtypes also seem functionally different, since MC_TC_ responds to various nonimmunological stimuli such as compound 48/80 and substance P, while MC_T_ does not [36]. Similarly, two major populations of mature mast cells have been described in rodents, defined mainly according to the tissue in which they reside. Connective tissue mast cells (CTMCs) are preferentially located around venules and nerve endings of skin, peritoneal cavity, and the digestive tract muscularis propria, whereas mucosal mast cells (MMCs) are mainly found in the intestinal and respiratory mucosa [23, 37]. Some of the factors involved in the development and proliferation of the CTMC subtype are SCF, NGF, and IL-4, while MMCs require SCF, IL-3, IL-9, IL-10, and TGF*β*. These latter factors are importantly secreted by T lymphocytes; hence, MMCs are usually considered T cell dependent [31, 32]. MMCs and CTMCs also differ in size and in their content of intragranular histamine, proteoglycans, and proteases. Specifically, MMCs are smaller than CTMCs, contain fewer granules with less histamine, and express mouse mast cell proteases-2 (mMCP-2), mMCP-4, mMCP-5, and mMCP-6, whereas CTMCs express primarily mMCP-1 and mMCP-2 [38, 39]. Additionally, MMCs contain proteoglycans with poorly sulfated glycosaminoglycans, such as chondroitin sulfate, while CTMCs contain highly sulfated glycosaminoglycans, such as heparin [40]. Taken together, these data support that there are different subtypes of mast cells, most likely fulfilling different functions and whose maturation is importantly shaped by their tissue location and the local stimuli provided by other resident immune cells [38, 41, 42].

Mast cell activation can lead to release of three distinct classes of bioactive molecules, depending on the type of stimuli and receptor involved: preformed mediators stored in their granules that are rapidly released (within seconds to minutes); de novo synthesized lipid mediators, prostaglandins, and leukotrienes (minutes); and a variety of cytokines and chemokines that are produced following their transcription and translation (hours). The most studied mechanism of mast cells activation is the response mediated through their high-affinity IgE receptor (FcεRI), which after their cross-linkage results in the rapid release of the granule content into the extracellular space, a process known as degranulation (Figure 1). This response also leads to the generation and release of the lipid inflammatory mediators derived from arachidonic acid, which are involved in leukocyte recruitment and activation, vasodilation, angiogenesis, and mitogenesis [33, 43].

Mast cell degranulation is also observed in many IgE-independent processes, such as degranulation induced by thrombin, IgG complexes, neuropeptides, and complement-derived anaphylatoxins [44–47]. Furthermore, mast cells have numerous other receptors on their plasma membrane, and the nature of the mast cell response is dependent on the stimulating ligand. For instance, mast cell activation by pathogen-associated molecular patterns (PAMPS) through Toll-like receptors (TLRs) triggers the differential and selective release of proinflammatory cytokines and chemokines with or without degranulation. For example, peptidoglycan (a TLR-2 ligand) can cause mast cell degranulation, while lipopolysaccharide (a TLR-4 ligand) does not. Moreover, a study showed that TLR-4 and TLR-6 elicit similar patterns of increased synthesis of GM-CSF, IL-8, and IL-10, whereas TLR-8 preferentially induces IL-8, MIP-1*α*, and TNF-*α*, and TLR-2 only IL-8 [47–50]. In addition to the rapid and massive release of granule content through exocytosis, there is significant evidence showing that mast cells can release granule compounds selectively by a process known as piecemeal degranulation, which involves vesicle transport from the granule to the plasma membrane, and it is the most prevalent form of mast cell mediator secretion identified in situ in several chronic diseases [51–56]. Taken together, these data highlight the phenotypic and functional plasticity of mast cells.

## 3. Overview of Mast Cells in Cancer

In 1878, Paul Ehrlich was the first to report the presence of mast cells in human tumors. Since then, there has been increasing evidence that mast cells, termed tumor-associated mast cells (TAMCs), infiltrate a variety of solid and hematological tumors. Examples of cancers with peritumoral or intratumoral high mast cells density are thyroid, stomach, pancreas, prostate, melanoma, and breast cancer [14, 57–60]. Puzzling, mast cells in these neoplasias have been reported as protumorigenic, antitumorigenic, or just as innocent bystanders [14]. Thus, increased accumulation of mast cells has been correlated with poor prognosis in gastric, pancreatic, and colorectal tumors [61–64]. Whilst in breast cancer, mast cell accumulation and function is still controversial (see Table 1).

The accumulation of TAMCs in different cancers may occur in response to various chemotactic factors secreted by tumor cells or immune cells in the tumor microenvironment. These can include SCF, monocyte chemotactic protein-1 (MCP-1), vascular endothelial growth factor (VEGF), angiopoietin 1 (Ang1), IL-8, CCL2, CXCL1, CXCL10, and osteopontin (OP), which in addition to recruit mast cell progenitors are also able to induce their maturation and activation [65–69]. Activated mast cells have been detected infiltrating angiosarcomas by electron microscopy; some of them exhibited anaphylactic degranulation while others exhibited piecemeal release [55]. However, tumor cells in close contact with activated mast cells did not show evidence of apoptotic or necrotic changes, thus concluding that it was unlikely that mast cells were battling cancer cells to contribute to the improvement of the clinical outcome [70]. This observation suggested that mast cell-mediator release by piecemeal could contribute to the selective release of protumorigenic mediators. Indeed, several protumorigenic functions for TAMCs have been reported, such as tumor cell proliferation, lymphatic and blood vessel formation, promotion of tumor cells invasion, and extravasation of diverse cytokine-producing cells [71–73]. TAMCs have also been shown to play a central role in angiogenesis of various types of tumors. In fact, mast cells can promote angiogenesis and lymphangiogenesis through the production not only of the classical proangiogenic mediators VEGF, fibroblast growth factor (FGF), IL-8, heparin, and metalloproteases but also of nonclassical factors, such as tryptase, chymase, and other serine proteases [65, 74, 75]. In melanoma, mast cell accumulation has been correlated with VEGF overexpression, increased neovascularization, enhanced tumor aggressiveness, and poor prognosis [76]. Moreover, mast cell production of tryptase correlates with local angiogenesis and tumor progression in skin tumors [71].

TAMCs can also promote tumor growth through the secretion of IL-8 and histamine, which function as chemotactic factors for immune cells and as tumor mitogens [77]. Furthermore, the production of different matrix metalloproteinases (e.g., MMP-9) and proteases (tryptase and chymase) by TAMCs can regulate the proteolysis of ECM proteins and disturb the physiological communication between stroma and epithelium, favoring detachment of cancer cells, migration, and invasion [61, 73, 78]. All these TAMC protumoral activities are in line with mast cell homeostatic functions related to wound healing and tissue repair [79–81]. On the other hand, TAMCs antitumor activities have also been documented. In this regard, using a murine model of intestinal carcinogenesis, one study demonstrated that mast cell-deficient mice developed more abundant and larger tumors than mast cell competent littermates [82]. Similarly, it has been reported that TAMCs can mediate tumor cell apoptosis through the production of IL-4, TNF, and reactive peroxides [83–85].

## 4. Mast Cells in Breast Cancer

**Figure 2:**
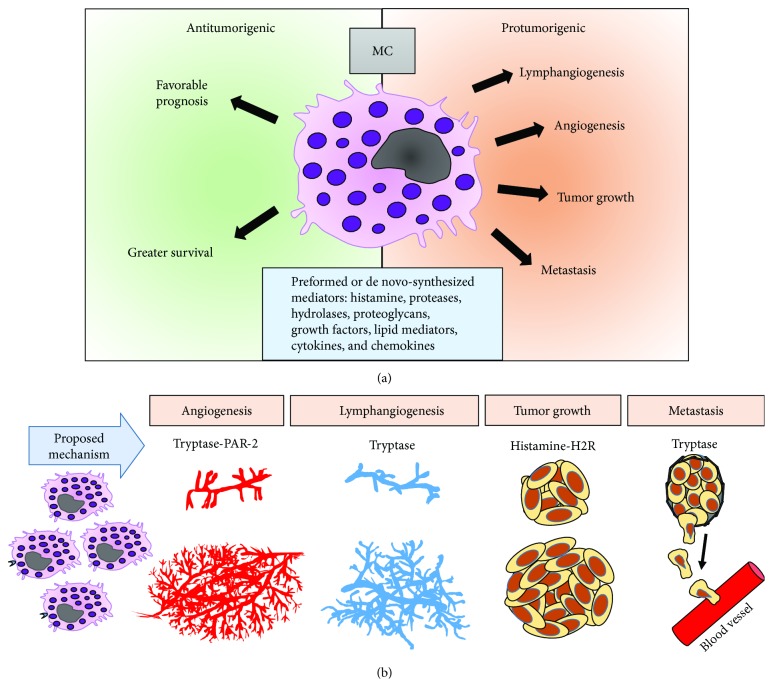
Role of mast cells in breast cancer. (a) The influence of mast cells in breast cancer prognosis is still a matter of discussion. Mast cells contain a great variety of bioactive components that may exert both pro- and antitumor effects. On the one hand, *in vitro* and *in vivo* studies support that mast cells exhibit protumor activity through promotion of lymphatic and blood vessel formation, tumor growth, and metastasis (orange right side). On the other hand, several population studies also associate mast cells with a greater survival and favorable prognosis (green left side). (b) Some bioactive molecules of mast cells documented to have protumorigenic effects are tryptase through its receptor PAR-2 and histamine through H2 receptor. The cancer processes in which these compounds have been associated are indicated.

Breast cancer is one of the most common causes of mortality and morbidity among women worldwide [86]. As in other cancers, mast cells are frequently observed in the tumor stroma of breast cancers, and their accumulation and prognostic significance have been a source of heated discussion with evidence of both pro- and antitumoral roles (Figure 2 and Table 1). To date, there is not yet a clear verdict on this ongoing debate.

### 4.1. Evidence of Mast Cell Antitumoral Function

Different clinical studies support a protective role for mast cells infiltrating breast tumors. Using a multivariate analysis, one study found that the presence of stromal mast cells was a positive prognostic factor, showing a strong correlation with survival curves, particularly for those cancers that still did not show evidence of lymph node invasion [87]. Compellingly, this study was further expanded to include a cohort of 4444 invasive breast cancer patients and a longer follow-up of up to 18.4 years. The conclusions reached were highly similar, with the authors proposing that mast cells could be used as a good prognostic marker, independent of age, tumor grade, tumor size, lymph node, and molecular subtype [88]. Naik et al. also found that higher numbers of mast cells in the axillary lymph nodes correlated with a better prognosis [89], although the pattern of distribution of mast cells in the different anatomic locations of the nodes was not different between the patients that survived and those that did not survive.

Perhaps, also very relevant is the specific participation of the MC_T_ and MC_TC_ subtypes for cancer progression. Unfortunately, this has only been addressed in one recent study in which no differences were found between both mast cell subtypes and prognosis; both MC_T_ and MC_TC_ cells were found infiltrating breast tumors, and both were associated with less aggressive cancer types (the luminal immunophenotype). Therefore, increased numbers of any of the mast cell subtypes correlated with a better disease prognosis [90]. Naik et al. also found a close association of mast cells with lymph node areas of high T cell density, similar to the location observed for T cell-dependent murine mucosal mast cells [89].

Another important factor that should be equated when studying the contribution of mast cells in breast cancer is their location within the tumor. Mast cells have been observed in either or both the intratumoral and peritumoral areas. More commonly, mast cells are almost exclusively found in the periphery of the tumor, frequently colonizing perivascular areas. Their potential role at these particular sites has been more difficult to elucidate, but the study of della Rovere et al. documented that peritumoral mast cells seemed to have a cytolytic activity against tumor cells [91].

### 4.2. Mast Cell Protumoral Function: Promotion of Angiogenesis and Metastasis

Similar to the evidence existing for other human cancers, several studies carried on with breast cancer patients have found a positive correlation between TAMCs and tumor angiogenesis. For example, one study found that high mast cell numbers correlated with increased microvascular density (MVD) in primary tumors [92]. However, the number of TAMCs did not correlate with other clinicopathological features of aggressive cancers, hampering the interpretation of their contribution to prognosis. Similarly, Samoszuk and Marech in 2003 and 2014, respectively, showed that TAMC numbers and tryptase levels in serum of breast cancer patients strongly correlated with MVD, supporting the involvement of mast cell-derived tryptase in tumor angiogenesis [93, 94]. Moreover, it has also been shown that microvessel counts increase in parallel to the number of tryptase-positive mast cells in lymph nodes from breast cancer patients and that their values are significantly higher in lymph nodes with micrometastasis compared with those without metastasis [95]. Therefore, it has been suggested that mast cells contribute, at least partially, to the micrometastasis that occur at early stages of tumor development and to the angiogenesis that supports it [92, 95].

Formation of lymphatic vessels or lymphangiogenesis is also a reliable predictor of lymph node metastasis [96, 97]. A recent study by Keser et al. found that mast cells were present in all invasive primary tumors and in the metastatic lymph nodes [98]. In previous studies, mast cells were generally observed in the stroma adjacent to the neoplastic cells and near vascular structures [87, 88, 99]. Confusingly, while mast cells were detected in all metastatic lymph nodes, not all enlarged lymph nodes with evidence of immune reactivity (reactive lymph nodes) showed evidence of their presence. Still, mast cell count was better correlated with metastatic lymph nodes than with reactive lymph nodes, which could indicate a specific mast cell role in metastasis of breast cancer cells. Although the study by Keser et al. did not find a correlation between mast cell density and disease-free/overall survival, the authors reported a significant link between lymphatic vessel density (LVD) and other poor prognostic parameters, such as tumor diameter, tumor volume, tumor nuclear grade, perineural invasion, metastatic lymph node count, and tumor stage [98]. Interestingly, LVD also correlated with a shorter period of disease-free survival. This apparently confusing data could be explained by a multifactorial influence on the clinical parameters measured in the study and/or by and indirect effect of mast cells in disease prognosis.

In support of the clinical correlation between cancer prognosis and tryptase serum levels, several *in vitro* studies have supported a direct effect of mast cell-derived tryptase on angiogenesis and lymphangiogenesis. Tryptase functions as an agonist of the proteinase-activated receptor-2 (PAR-2) in vascular endothelial cells stimulating their proliferation [100, 101]. Tryptase also induces angiogenesis by releasing stored angiogenic factors bound to the ECM, such as cytokines and metalloproteinases [102–104]. A study in 2009 showed that the peritumoral levels of tryptase augmented with the grade of the tumor, and this correlated positively with lymph node metastasis [104]. In agreement, MDA-MB-231 cells, a breast cancer cell line, increased migration and invasion in response to tryptase in transwell assays [104].

Concerning the tumor location of mast cells, one study found that intratumoral mast cells were better associated with lymphatic and perineural invasion, and this was an adverse prognostic parameter [98]. Contrary to della Rovere et al.'s study, indicating a potential positive role for peritumoral mast cells, other studies have found that these cells secrete proteases that facilitate vascular invasion and accelerate metastatic spread [92, 95, 98]. Thus, peritumoral mast cells seem to contribute to breast cancer progression. Fakhrjou et al. also found a positive association between the number of mast cells and the histopathological grade of the disease, particularly in invasive ductal carcinoma [99].

Experimental data using mast cell-deficient mice have also provided a strong support for a positive correlation between mast cells in mammary tumors and metastasis. In the study by He et al., mast cell-deficient mice (Kit^W-sh/W-sh^), a c-Kit knockout strain, was crossed with mice that spontaneously develop breast cancer (PyMT strain). Although the number and the onset of tumors was not affected in the offspring, the size of the tumor and their metastatic potential were significantly reduced in the c-Kit-deficient mice compared with their littermate controls [105]. Moreover, histological examination of tumors revealed a marked decreased in angiogenesis, thus supporting the fact that mast cells are not as important for tumor initiation as they are for tumor progression and that their contribution is strongly due to their ability to promote tumor vascularization.

## 5. Mast Cells Are Potential Targets for Anticancer Therapy

Experimental studies in mice have suggested that mast cell inhibitors could reduce the number and activity of the cells in certain types of cancers improving disease outcomes [106]. For instance, in murine models of prostate adenocarcinoma, treatment with cromolyn (sodium cromoglicate), a well-known mast cell degranulation inhibitor, blocked prostate tumor growth. Paradoxically, treated mice developed highly malignant neuroendocrine cancers, a fatal collateral event that should be more deeply studied before proposing the use of cromolyn or the targeting of mast cells as therapy [106]. On the other hand, in a preclinical study involving pancreatic cancer patients treated with the drug masitinib, a tyrosine-kinase inhibitor that has inhibitory activity against c-Kit compromising mast cell survival, it was shown that patients receiving a combination of masitinib plus standard chemotherapy had an increased survival compared with patients receiving chemotherapy alone [107]. However, it is important to note that the study did not distinguish whether the increased survival was directly related to mast cell activity.

Other *in vitro* and *in vivo* studies using mast cell stabilizers or mast cell-depleting agents have shown controversial results. For instance, depletion of mast cells with imatinib enhanced tumor growth in a murine model of breast carcinoma [108], supporting an antitumoral role for mast cells. In agreement, mice treated with cromolyn showed mammary tumors with extensive hypoxic hemorrhagic regions and clots, which were not observed in the control group, suggesting that mast cells play an important role in inhibiting blood clotting and maintaining blood perfusion in breast cancer, probably through secretion of heparin, plasminogen activator, chymase, and tryptase [94].

Histamine, one of the most important components of mast cell granules, has been shown to be critical for development of the normal rat mammary gland [110]. Likewise, histamine has been implicated in promoting tumor cell proliferation and enhancing growth of experimental mammary carcinomas, particularly acting through H2 receptors, and treatment with H2 receptor antagonists significantly inhibited tumor cell proliferation and tumor growth [109, 110]. However, a human clinical trial testing the H2 receptor antagonist cimetidine (Tagamet), found no relationship between the preoperative drug administration and breast cancer growth [111].

## 6. Conclusions

We have recently understood that tumor infiltration by immune cells is not necessarily a good sign for defense and protection. Tumor-associated macrophages have been the torch bearers to help us recognize how the immune system contributes to cancer progression often as early as precancerous stages. Although we have learned a good deal about the role of mast cells in cancer, we still lag behind and are far from understanding their potential protective or harmful influence. This, in spite of the wide range of bioactive molecules inherent to the activity of mast cells, potentially places them in a broad number of cancer-associated biological processes.

Most studies agree that breast tumors are infiltrated by mast cells. However, there is conflicting data about the meaning of that observation in terms of disease prognosis. The source of discrepancy may have different origins, from technical to biological, for instance, the markers and methods used to identify and count mast cells or the clinical parameters used to give correlative associations. Furthermore, cancer in general, and breast cancer in particular, is a highly heterogeneous disease with a great variety of genetic/histological/clinical subtypes, with each subtype also exhibiting a high heterogeneity within itself. It is possible that mast cell contribution, either positive or negative, is specific to certain breast cancer subtypes or that mast cells and the inflammatory microenvironment influence each other independently of other histological features. Indeed, mast cells are highly reactive cells that express a great variety of receptors and respond to a great variety of stimuli influencing their maturation, density, and activation, polarization into different subtypes, content of biomolecules, and immediate or persistent release mechanisms [112–114]. Although two mast cell-derived factors, tryptase and histamine, seem to perform a protumorigenic role in breast cancer, there are multiple other mast cell biomolecules for which we do not know much about their possible participation in cancer progression. For instance, arachidonic acid-derived lipid mediators, which are also involved in angiogenesis and mitogenesis. More work is needed to clarify the role of mast cells in breast cancer and for a better understanding of the mechanisms of mast cell communication with tumor cells and other immune cells within the tumor stroma.

## Figures and Tables

**Table 1:** Studies analyzing the participation of mast cells in breast cancer.

Study type	BC specimen	MC detection method	Prognosis in BC	Association	Ref.
D/C/E	Tumor tissue and blood ^∗^xenotransplanted mice	Tryptase	Positive	Decreased blood clotting and hypoxia	[94]
D/C	Tumor tissue	c-kit (CD117)	Positive	Greater survival	[95]
D/C	Tumor tissue from IDC	c-kit (CD117)	Positive	Greater survival	[87]
D/C	Tumor tissue	Giemsa and Alcian blue	Positive	BC subtype	[88]
D/C	Lymph nodes	Toluidine blue	Positive	Greater survival	[91]
D/C	Tumor tissue from IDC	Tryptase and chymase	Positive	BC subtypes	[89]
D/C	Sentinel lymph nodes	Tryptase	Negative	Angiogenesis and micrometastasis	[90]
D/C	Tumor tissue	Tryptase	Negative	Angiogenesis	[92]
D/C	Tumor tissue and sera to measure tryptase levels	Tryptase	Negative	Angiogenesis	[93]
D/C	Tumor tissue and lymph nodes from IDC patients	Toluidine blue	Negative	Angiogenesis	[98]
D/C	Tumor tissue from IDC	Toluidine blue	Negative	BC grade	[99]
D/C/E	Benign growths and tumor tissues Cell line treated with tryptase	Tryptase	Negative	BC grade and metastasis	[104]
E	^∗^Mast cell-deficient BC-prone mice	Toluidine blue	Negative	Progression, metastasis, and angiogenesis	[105]
C/E	Tumor tissue from cimetidine treated patients	Toluidine blue	None	None	[111]

BC: breast cancer; IDC: invasive ductal carcinoma; D: descriptive study; C: correlative; E: experimentally tested; positive: antitumoral role; negative: protumoral role. ^∗^Studies also performed in mice.
